# Shame and guilt activations: Associations and sociodemographic differences among ploysubstance abusers

**DOI:** 10.3389/fpsyt.2022.1021876

**Published:** 2022-10-21

**Authors:** Najam ul Hasan Abbasi, Mujahid Iqbal, Yu Yan, Sumaira Mubarik, Muhammad Nadeem, Mehmet Behzat Turan, Romana Younas

**Affiliations:** ^1^Department of Education Sciences, Mianyang University, Mianyang, China; ^2^Department of Psychology, School of Philosophy, Wuhan University, Wuhan, China; ^3^Department of Epidemiology and Biostatistics, School of Public Health, Wuhan University, Wuhan, China; ^4^Department of Psychology, University of Lahore, Lahore, Punjab, Pakistan; ^5^Faculty of Sport Science, Erciyes University, Kayseri, Turkey; ^6^School of Psychology, University of Chinese Academy of Sciences, Beijing, China

**Keywords:** guilt-activation, shame-activation, inpatient treatment, polysubstance abusers, healthy individuals

## Abstract

**Objectives:**

Shame and guilt in polysubstance abusers are still understudied despite their significance in substance use disorders (SUD). The goal of the current study is to develop a better understanding of how shame and guilt interact among polysubstance abusers who are receiving residential treatment.

**Methods:**

The sample of two hundred four males with SUD admitted to five rehabilitation centers from two cities in Pakistan participated in this study. For comparison, 215 age-matched healthy individuals were recruited (control). All participants reported their scores on the state shame and guilt scale (SSGS) and demographic form. A cross-sectional study design was adopted.

**Results:**

The group with SUD reported greater activations on SGSS (*r* = 0.79, *p* < 0.001) as compared to healthy (control) individuals (*r* = 0.48, *p* < 0.001). Further, multivariate analysis indicated that people with SUD who were of older age, unemployed, living in a nuclear family system, with a higher level of education, and low income, experienced higher levels of shame and guilt. Multinomial logistic regression analysis revealed that people with SUD in the age group 41–60 years (OR 5.2, 95%CI 2.4–6.8), unemployed (OR 4.4, 95%CI 3.2–4.7), nuclear family system (OR 5.9, 95%CI 4.5–6.4) and low monthly income group (OR 5.4, 95%CI 3.5–5.8) had a significantly high risk of shame and guilt than the control group.

**Conclusion:**

Findings of the current study indicate an association between shame and guilt activation and SUD. These results suggest that polysubstance users may benefit from therapeutic interventions to avoid a generalization of shame and guilt toward their substance use. Reducing shame and guilt should be considered a priority in treating adults with multiple SUD.

## Introduction

There is evidence that individuals with a history of addictive behaviors experience excessive self-conscious emotions, and shame and guilt can be especially challenging (1–3). Shame is often confused with guilt. Shame is more related to the self (e.g., “I am a bad person”) than the behavior (4). It is a painful feeling that occurs due to negative evaluations of the self and fear of negative evaluations by others about the self (5). Whereas guilt is more focused on behavior (e.g., “the behavior I have committed is bad”).

Toxic shame and chronic guilt are major reasons for all addictive behaviors (6). A study found a positive association between shame and guilt activation in alcohol dependence. The author claimed that people with substance use disorder (SUD) reported a higher level of guilt than those who didn't use drugs (7). Additionally, shame can be a barrier to recovery from the treatment of addictive behaviors (8). Shame predicts a tendency to relapse, the severity of relapse, and a decline in mental and physical health.

Research conducted on alcoholics presented that shame and guilt activation were positively associated (9). However, guilt is positively correlated with protective behavior strategies, and shame is negatively associated with alcohol consumption. One other study suggested that university drinkers who took more alcohol and experienced more alcohol-related problems scored higher on the shame. On the contrary, lower scores were reported on the guilt (10). Regarding sociodemographic factors, a study on aging and self-conscious emotions in a non-clinical sample proved that shame and guilt are usually reported by older adults (11). A study in Vietnam reported that unemployed men with SUD, faced more stigmatization and had a sense of shame and guilt activation (12).

Based on the researchers' initial review of related literature, it was found that limited studies have been conducted on this topic in Pakistan among inpatient poly-substance abusers. Some researchers have claimed shame and guilt are detrimental to treatment-seeking behaviors (4, 7, 8, 13), and others proved these are important to make reflections on wrongdoings (14, 15). The relationship between shame and guilt activations is still unclear in inpatient polysubstance abusers. Thus, more studies are needed to investigate the relationship between the two constructs.

Thus, the current research was designed (a) to explore the relationship between shame and guilt activations among inpatient polysubstance abusers and (b) to investigate the difference between shame and guilt activations concerning sociodemographic factors. It was hypothesized that there would be a strong positive correlation between shame and guilt activations in polysubstance abusers. Further, it was expected that there might be significant differences in shame and guilt activations in sociodemographic variables.

## Materials and methods

### Participants

The current study adopted cross-sectional research design. A sample of 204 males, polysubstance abusers with ages ranging from 18- 65 years (*Mean* = 31, *SD* = 4.6) were recruited in five rehabilitation centers in two cities of Pakistan (Lahore and Mirpur Azad Kashmir). To receive services at the hospital participants had to meet the criteria (presented at least six or more symptoms, ensuring the severity) of SUD according to the diagnostic and statistical manual of mental disorders (DSM-V). It has already been defined by Flanagan (4) that people with SUD often report feelings of shame and guilt. So, all participants in the addiction group were polysubstance users and were receiving residential treatment. All patients had recovery of at least 21 days and were recruited during their third week of detoxification. They had no other psychiatric disorders. The presence of such other psychiatric diseases is evaluated through an in-depth psychiatric investigation conducted by a qualified psychiatrist and clinical psychologist. For comparison purposes, a control group of 215 males aged 18–65 years (*Mean* = 30, *SD* = 3.9), who were not using treatment services for alcohol and drug problems and were free of any psychiatric disorder, were recruited for the study. The demographic characteristics of the control group were matched, as much as possible, with the group of SUD. Divisions of the sample are given in Table 1.

**Table 1:** Sociodemographic characteristics between the group with SUD and control group.

**Variables**	**Group with SUD** **(*n*=204)**		**Control group** **(*n* = 215)**		***P*-value**
	***n***	***%***	***n***	***%***	
Age (mean ± SD)	31 ± 4.6		30 ± 3.9		0.471
Shame level (mean ± SD)	4.8 ± 0.25		3.32 ± 0.71		<0.001
Guilt level (mean ± SD)	4.7 ± 0.24		2.9 ± 0.55		<0.001
**Education**					
High school	43	21.1	6	2.8	<0.01
College level	95	46.6	33	15.3	
Graduate	49	24.0	84	39.1	
Post-graduate	17	8.3	92	42.8	
**Occupation**					
Students	10	4.9	61	28.4	<0.01
Employed	60	29.4	136	63.3	
Unemployed	134	65.7	18	8.4	
**Family system**					
Joint	148	72.5	147	68.4	0.204
Nuclear	56	27.5	68	31.6	
**Monthly income**					
Less than 30K	131	64.2	68	31.6	<0.001
30–60K	53	26.0	68	31.6	
61–90K	9	4.4	38	17.7	
Above 90K	11	5.4	41	19.1	
**Marital status**					
Married	114	55.9	72	33.5	<0.001
Single	90	44.1	143	66.5	

Comparison of qualitative variables' proportion between the group with SUD and control group was performed using chi-square test, and quantitative variables' mean between groups was compared using t-test. Coding of Shame and guilt level (1 = Not feeling this way at all, 2 = Feeling this way a little, 3 = Feeling this way somewhat, 4 = Feeling this way quite a bit, 5 = Feeling this way very strongly).

### Procedure

The ethics board committee of the Department of Psychology, Wuhan University, China, approved the study. Participants were approached in five rehabilitation centers and debriefed about the study procedures. Once they understood the study protocols, they signed the informed consent and filled in the questionnaire and demographic sheet.

The control group was recruited via the snowball sampling technique. Data was collected online by social web-based applications such as WhatsApp, Messenger, WeChat, and Facebook. The survey was created using the online survey tool at https://docs.google.com. The research purpose was explained and the participants in an online survey signed informed consent. It was mentioned that only males above 18 years of age who didn't take alcohol and drugs could participate in this research. The participants confirmed that they had not experienced addiction or accessed services for addiction. This study was not time-bound; however, completing the questionnaires takes 10–15 min. Participants weren't compensated for taking part in this study. All Data were collected from August to December 2021.

### Inclusion and exclusion criteria for the group with SUD

All participants included in the group with SUD were males over the age of 18 years, diagnosed with polydrug addiction, receiving residential treatment, in recovery of at least 21 days, and free from other psychiatric disorders. The study excluded patients with a single drug use, outpatient treatment, a neurological problem, or a chronic illness and patients who were unwilling to participate.

### Inclusion and exclusion criteria for the control group

All participants in the control group were males over 18, without a history of substance abuse or psychiatric disorders and any need for treatment. Participants whose ages did not match, those who had a mental disease, those undergoing treatment for psychiatric problems, and were unwilling to participate were excluded from the study.

### Measures

One author physically visited the hospitals/rehab clinics and explained each questionnaire item to the participants in Urdu. This researcher is a Ph.D. scholar and lecturer at a university. He is also fluent in the English language and a native Urdu speaker.

### Demographic form

We developed a 6-item demographic form to include in the survey packet. Questions addressed age, education, occupation, family system, monthly income, and marital status. The age of participants was asked as a quantitative variable. Education, occupation, family system, monthly income, and marital status were categorical variables.

### State shame and guilt scale

The 10 items of two subscales from the state shame and guilt scale developed by Marschall et al. (16), were used by both the addiction and control groups. The SSGS has already been used in SUD literature (17) and in non-clinical populations (18). The original scale comprises 15 items and 3 subscales (shame, guilt, and pride). Each subscale consists of 5 items. Example items were “I feel like I am a bad person.” and “I cannot stop thinking about something bad I have done.” SSGS is based on a 5-point Likert-type scale with response categories including 1 = *Not feeling* this way at all to 5=*Feeling this way very strongly*. Minimum-maximum scores for each subscale are 5 to 25. As this was a state scale, participants rated their responses on the scales based on how they were feeling at the moment. The scores on the scale were interpreted as the lower and higher score. Cronbach's alpha for the two sub-scales were strong: Shame = 0.89 and guilt = 0.80.

### Data analysis

Descriptive statistics were used to clarify the detail of the demographic sample. Pearson correlation coefficient was applied to quantify the relationship between shame and guilt. Further, Spearman rank correlation was performed to investigate the correlation of sociodemographic characteristics with shame and guilt scores among group with SUD and control. The differences in qualitative (demographic) variables (age, education, occupation, family system, family monthly income, and marital status) between the addiction and control group were examined, a Chi-square test was performed, *t*-test was used to compare the shame and guilt mean score between two groups. To measure the variability of shame and guilt across the group and sociodemographic factors a Multivariate two-way MANOVA was performed.

Additionally, the association of sociodemographic factors with different levels/degrees of shame and guilt was tested using Multinomial logistic regression analysis. Dummy variables 1 to 5 were assigned to shame/guilt levels, and four models were estimated by keeping the level-1(Not feeling this way at all) as the reference group. All models were estimated separately for the group with SUD and control. Results were interpreted using the Odds ratio (OR) with a 95% confidence interval (CI) for each independent variable.

Effect sizes were calculated using partial eta squared and interpreted using (19) guidelines for small ($\eta_{\text{p}}^{2}$ = 0.01), medium ($\eta_{\text{p}}^{2}$ = 0.06), and large effect sizes ($\eta_{\text{p}}^{2}$ = 0.14). The *p-*value <0.05 was considered statistical significance for all analyses. All analyses were performed using SPSS version 26 IBM.

## Results

The study was conducted on age-matched participants between the group with SUD and the control, with the mean age of the group with SUD (*M* = 31, *SD* = 4.6) and the control (*M* = 30, *SD* = 3.9). The overall mean shame and guilt level for the group with SUD was about 2 times higher than the control, followed by the mean level of shame (*M*= 4.8, *SD* = 0.25) and mean guilt level (*M* = 4.7, *SD* = 0.24) for the people with SUD. The detailed descriptive statistics for the level of education, employment status, family system, and sample group characteristics can be seen in Table 1.

**Figure 1:**
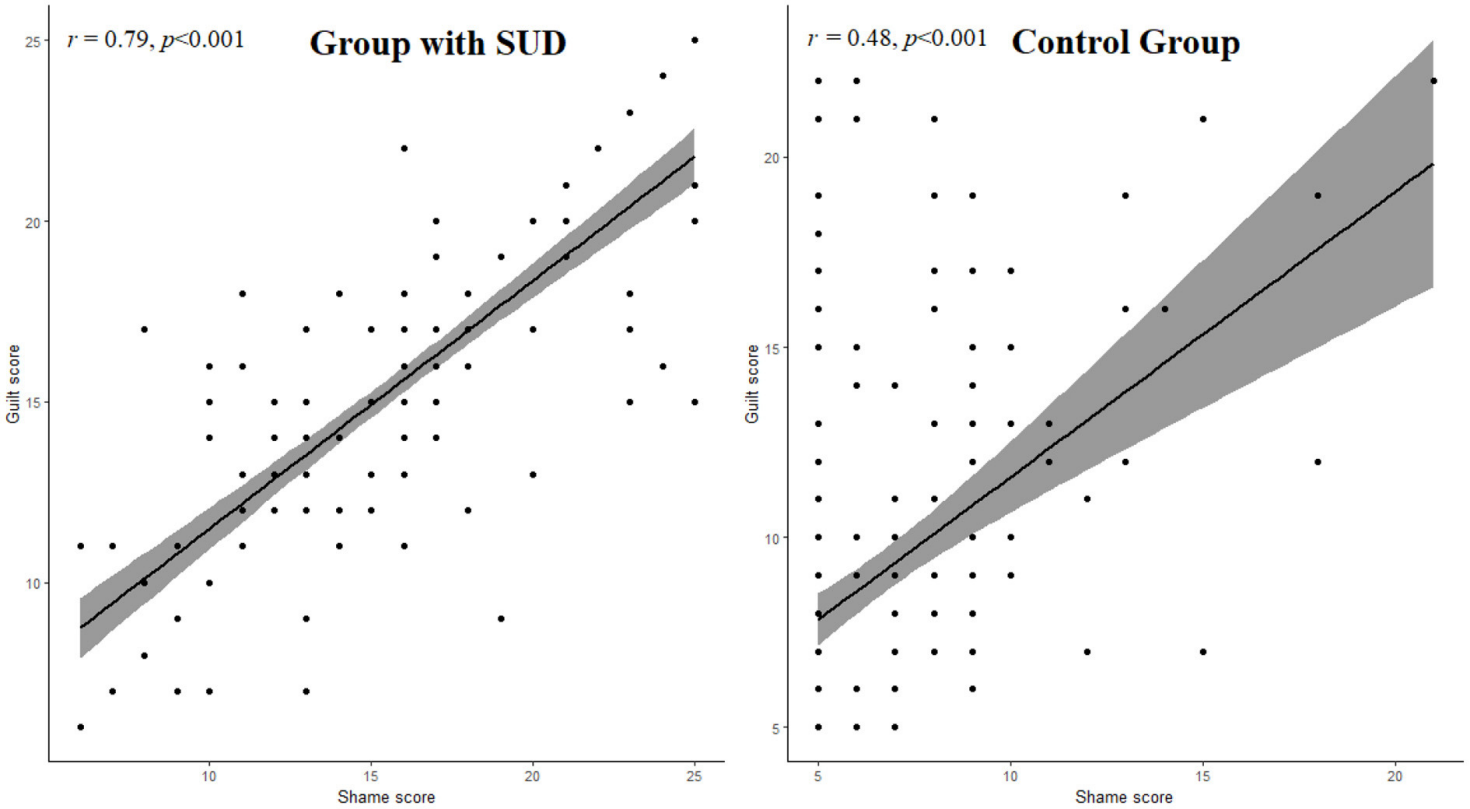
Relationship between shame and guilt in the group with SUD and control group. *r*, Pearson correlation coefficient.

The Pearson correlation coefficient between shame and guilt score was performed separately for both group with SUD and control individuals. We observed a significantly high correlation between shame and guilt scores among people with SUD (*r* = 0.79, *p* < 0.001) as compared to control (see Figure 1).

Further, the correlation of sociodemographic factors with shame and guilt scores of people with SUD and control individuals was computed, which revealed that education level was moderately correlated with guilt activation in the group with SUD (*r* = 0.26; *p* < 0.05) and had a linear correlation in the control group (*r* = 0.22; *p* < 0.05). Table 2 below shows that occupation was strongly correlated with shame-activation and guilt-activation in the group with SUD. At the same time, these variables were also linearly correlated in the control group. Results show that the family system had a significant positive correlation with shame and guilt-activation in the group with SUD. In contrast, the control group showed a linear correlation for these variables. Remarkably, family monthly income was negatively correlated with shame-activation and guilt-activation in the group with SUD, as shown in Table 2.

**Table 2:** Correlation of sociodemographic characteristics with shame and guilt-activation among group with SUD and control group.

	**Group with SUD**		**Control group**	
	**Shame activation**	**Guilt activation**	**Shame activation**	**Guilt activation**
	**r_s_**	**r_s_**	**r_s_**	**r_s_**
Age	0.07	0.09	0.04	0.01
Education	0.19	0.26*	0.14	0.22*
Occupation	0.56*	0.49*	0.41*	0.37*
Family system	0.45*	0.50*	0.39*	0.41*
Monthly income	−0.48*	−0.36*	−0.19	−0.23*
Marital status	0.03	0.06	0.05	0.07

^*^Indicate significant correlation coefficient (r_s_) at p < 0.05. r_s_, Spearman rank correlation coefficient; Monthly income = Family Monthly income.

For mean comparison by sociodemographic factors between the group with SUD and control was performed using multivariate two-way MANOVA. Results show significant mean differences in shame and guilt across age groups between the group with SUD and control. Participants from the group with SUD scored higher on shame and guilt levels in all age groups compared to the control group. By education level comparison, results revealed significant mean differences between groups. Participants in SUD group reported high mean scores compared to the control. Furthermore, findings revealed a significant multivariate main effect for occupation between the group with SUD and the control group. This partial eta square value indicates a large effect size. Comparison over occupation categories revealed that unemployed participants got high scores than other occupation ranks on shame and guilt in the group with SUD. Moreover, findings showed significant mean differences in both groups concerning the family system. The group with SUD scored higher on shame and guilt, than control group (Wilks' λ = 0.42, *F* (2.414) = 283.22, *p-*value < 0.001, $\eta_{\text{p}}^{2}$ = 0.14). Moreover, participants from the nuclear family system reported high scores on shame and guilt among people with SUD. When considering family monthly income, the finding revealed that participants from the group with SUD scored higher on shame and guilt than those from the control group.

Furthermore, we observed that people with SUD having low income reported greater shame and guilt score than other income levels. There were significant mean differences in marital status between the group with SUD and the control. People with SUD having single marital status got higher scores on shame and guilt than the control group. No significant differences were found in multivariate interactions between group and sociodemographic factors on shame/guilt activation (see Table 3).

**Table 3:** Mean comparison of group with SUD and control group by sociodemographic factors on shame and guilt using multivariate analysis.

		**Group with SUD**		**Control group**		**Multivariate tests two-way MANOVA**	
**Variable**		**Shame score**	**Guilt score**	**Shame score**	**Guilt score**	Wilks' lambda λ (F_P−*value*_)*	Partial eta squared (η^2^)*
Age group	Below 20(yrs.)	17.75	16.75	11.90	12.50	0.96 (0.06)	0.01
	20-40(yrs.)	18.84	17.69	13.88	14.53		
	41-60(yrs.)	18.90	17.36	12.67	12.09		
	Above 60(yrs.)	19.41	18.00	14.93	15.07		
	**Wilks' lambda** λ **(F**_**P−*value***_)**	0.59 (0.01)					
	Partial eta squared (η^2^)**	0.05					
Education	High school	18.35	19.14	15.67	14.33	0.97 (0.05)	0.09
	College	17.37	18.21	15.21	15.73		
	Graduate	19.61	20.18	15.13	16.89		
	Post-graduate	18.65	17.82	12.55	14.00		
	**Wilks' lambda** λ **(F**_**P−*value***_)**	0.56 (*P* < 0.001)					
	Partial eta squared (η^2^)**	0.08					
Occupation	Students	17.30	16.60	12.89	14.43	0.78 (P < 0.001)	0.15
	Employed	19.70	17.33	15.44	16.41		
	Unemployed	23.16	21.81	17.06	18.89		
	**Wilks' lambda** λ **(F**_**P−*value***_)**	0.65 (0.02)					
	Partial eta squared (η^2^)**	0.13					
Family system	Joint	20.21	19.32	14.10	16.21	0.46 (P < 0.001)	0.18
	Nuclear	23.01	22.41	16.01	18.10		
	**Wilks' lambda** λ **(F**_**P−*value***_)**	0.42(*P* < 0.001)					
	Partial eta squared (η^2^**	0.14					
Family monthly income	Below 30K	22.67	20.27	17.84	16.96	0.68 (0.04)	0.19
	30–60K	20.13	19.02	15.96	14.88		
	61–90K	17.33	16.00	12.42	13.92		
	Above 90K	15.18	14.64	10.32	11.84		
	**Wilks' lambda** λ **(F**_**P−*value***_)**	0.53 (0.03)					
	Partial eta squared (η^2^)**	0.16					
Marital status	Married	20.79	19.54	16.54	15.51	0.88 (0.08)	0.03
	Single	21.12	20.59	17.78	16.41		
	**Wilks' lambda** λ **(F**_**P−*value***_)**	0.41 (0.01)					
	Partial eta squared (η^2^)**	0.12					

^*^The test statistic p-value for mean comparison of sociodemographic factors; **The test statistic p-value for mean comparison between groups.

A Multinomial logistic regression analysis was performed to investigate the association of sociodemographic factors with different levels of shame and guilt. Results indicated that people with SUD in the higher age group (41–60 yrs.) were significantly associated with a high risk of shame in model-2 to model-4 compared to the reference group (Not feeling this way at all), i.e. ORs (95% Cis), 2.5 (1.8–3.6) for model-2, 4.3 (2.7–5.4) for model-3 and 5.2 (2.4–6.8) for model-4. As for other demographic variables, the participants belonging to unemployed, nuclear family system, and low monthly income groups had a significantly high risk of shame and guilt in the last three models as compared to the reference group among both groups with SUD and control individuals (see Tables 4, 5).

**Table 4:** Association of socioeconomic factors with different levels of shame in group with SUD and control group.

	**Model**−**1** **Feeling this way a little**		**Model**−**2** **Feeling this way somewhat**		**Model**−**3** **Feeling this way quite a bit**		**Model**−**4** **Feeling this way very strongly**	
	**OR (95% CI)**		**OR (95% CI)**		**OR (95% CI)**		**OR (95% CI)**	
**Predictors**	**Group with SUD**	**Control group**	**Group with SUD**	**Control group**	**Group with SUD**	**Control group**	**Group with SUD**	**Control group**
**Age group**								
Less than 20 years	1.7 (0.5–2.3)	0.7 (0.5–2.0)	2.1 (0.8–3.1)	1.1 (0.9–2.1)	2.7 (0.8–3.2)	1.3 (0.6–1.7)	3.4 (0.8–4.1)	2.1 (0.7–3.0)
20–40 years	1.5 (0.5–2.0)	0.3 (0.2–3.0)	2.3 (0.4–3.1)	1.5 (0.8–2.1)	3.2* (1.3–4.2)	1.6 (0.3–2.2)	4.2* (2.8–5.3)	2.2 (0.9–3.2)
41–60 years	2.1 (0.5–2.5)	0.8 (0.6–2.5)	2.5* (1.8–3.6)	1.7 (0.5–2.4)	4.3* (2.7–5.4)	1.8 (0.5–2.3)	5.2* (2.4–6.8)	3.1 (0.7–4.1)
Above 60 years	1.8 (0.7–3.5)	1.6 (0.3–2.1)	1.4 (0.8–2.1)	0.9 (0.4–2.3)	2.9 (0.5–3.4)	0.6 (0.3–2.4)	3.2 (0.9–4.0)	1.5 (0.5–2.5)
**Education level**								
High school	2.3 (0.8–2.9)	0.7 (0.4–2.3)	3.2 (0.7–4.1)	1.8 (0.5–2.7)	3.1 (0.9–3.8)	2.3 (0.5–3.1)	3.6 (0.4–4.4)	2.5 (0.9–3.3)
College	2.5 (0.7–1.8)	0.8 (0.5–1.4)	3.1 (0.9–3.9)	1.2 (0.6–2.1)	4.0 (0.9–4.5)	2.5 (0.7–2.9)	4.3 (0.6–4.8)	3.6 (0.4–4.1)
Graduate	2.8 (0.8–3.1)	1.4 (0.3–2.0)	3.4 (0.4–4.3)	2.9 (0.7–3.4)	4.2 (0.9–4.8)	2.9 (0.3–3.4)	5.2 (0.8–5.6)	4.8 (0.5–5.0)
Post–graduate	3.1 (0.7–3.5)	2.3 (0.6–2.7)	3.6 (0.9–4.0)	2.1 (0.5–2.6)	4.5 (0.6–5.0)	3.2 (0.8–3.7)	3.4 (0.9–3.8)	2.4 (0.5–3.2)
**Occupation**								
Students	2.1 (0.3–2.5)	1.6 (0.4–2.3)	2.4 (0.7–2.7)	1.1 (0.1–1.5)	3.2 (0.9–3.5)	2.5 (0.8–2.8)	3.9 (0.6–4.2)	2.4 (0.5–2.7)
Employed	2.3 (0.8–2.6)	0.9 (0.3–1.2)	2.7 (0.8–2.9)	1.7 (0.3–2.0)	3.4 (0.7–3.7)	1.2 (0.4–1.5)	3.5 (0.9–3.8)	2.6 (0.8–2.9)
Unemployed	2.8 (0.9–3.1)	0.5 (0.1–0.8)	3.4* (2.5–3.8)	1.9 (0.5–2.2)	3.9* (2.8–4.3)	2.1 (0.7–2.5)	4.4* (3.2–4.7)	3.2 (0.9–3.5)
**Family System**								
Joint	2.3 (0.8–2.9)	1.8 (0.5–2.3)	3.3 (0.9–3.9)	2.1 (0.6–2.8)	4.3* (3.2–4.8)	3.5* (2.6–4.2)	5.6* (4.5–5.9)	4.0* (3.5–4.5)
Nuclear	2.6 (0.9–2.9)	1.9 (0.7–2.2)	3.8* (2.7–4.7)	2.9 (0.9–3.3)	5.1* (4.1–5.5)	4.2* (3.5–4.7)	5.9* (4.5–6.4)	4.6* (3.8–4.9)
**Income**								
Below 30K	2.4 (0.8–2.7)	1.1 (0.5–1.5)	3.6* (2.5–4.2)	2.3 (0.4–2.7)	4.3* (2.5–4.8)	2.7 (0.9–3.1)	5.4* (3.5–5.8)	3.8 (0.7–3.2)
30–60K	3.1 (0.9–3.5)	1.5 (0.6–1.8)	2.4 (0.7–1.7)	1.1 (0.4–1.5)	3.2 (0.8–3.7)	1.6 (0.5–2.1)	4.8* (3.7–5.1)	3.1 (0.9–3.5)
61–90K	2.5 (0.7–2.8)	1.3 (0.4–1.6)	2.0 (0.8–2.5)	1.8 (0.7–2.2)	2.9 (0.9–3.4)	2.8 (0.6–3.2)	3.9 (0.8–4.2)	2.7 (0.7–3.1)
Above 90K	2.8 (0.8–3.1)	1.5 (0.3–1.8)	1.7 (0.5–2.2)	1.4 (0.3–1.8)	1.8 (0.7–2.3)	1.5 (0.6–1.8)	2.6 (0.9–3.1)	2.2 (0.7–2.6)
**Marital status**								
Married	1.7 (0.5–2.2)	1.5 (0.5–1.8)	2.8 (0.8–3.1)	2.4 (0.7–2.7)	4.3 (0.9–4.7)	3.2 (0.8–3.6)	5.2 (0.9–5.5)	3.9 (0.9–4.2)
Single	1.8 (0.6–2.3)	1.7 (0.7–2.4)	3.7 (0.9–4.1)	2.5 (0.7–2.8)	4.7 (0.9–5.1)	3.5 (0.8–3.8)	5.4 (0.9–5.7)	4.3 (0.7–4.6)

Reference category: Not feeling this way at all; ^*^Indicates significant at 0.01 level.

**Table 5:** Association of socioeconomic factors with different levels of guilt in group with SUD and control group.

	**Model**−**1** **Feeling this way a little**		**Model**−**2** **Feeling this way somewhat**		**Model**−**3** **Feeling this way quite a bit**		**Model**−**4** **Feeling this way very strongly**	
	**OR (95% CI)**		**OR (95% CI)**		**OR (95% CI)**		**OR (95% CI)**	
**Predictors**	**Group with UD**	**Control group**	**Group with SUD**	**Control group**	**Group with SUD**	**Control group**	**Group with SUD**	**Control group**
**Age group**								
Below 20 years	1.8 (0.5–2.0)	0.9 (0.5–2.1)	2.4 (0.8–2.8)	1.5 (0.9–1.9)	2.8 (0.8–3.1)	1.7 (0.6–2.0)	3.5 (0.8–4.1)	2.4 (0.7–2.8)
20–40 years	1.9 (0.6–2.1)	1.2 (0.4–1.5)	2.5 (0.5–3.1)	1.7 (0.8–2.1)	3.5* (2.3–4.1)	2.2 (0.9–2.5)	4.5* (3.5–4.9)	3.1 (0.9–3.5)
41–60 years	2.3 (0.7–2.7)	1.8 (0.6–2.2)	2.8* (2.3–3.5)	1.9 (0.7–2.3)	4.5* (3.5–4.9)	2.5 (0.8–2.8)	5.5* (4.5–5.8)	3.3 (0.9–3.7)
Above 60 years	2.8 (0.8–3.1)	2.3 (0.8–2.5)	2.5 (0.9–2.8)	2.3 (0.7–2.7)	3.9 (0.9–4.2)	2.6 (0.8–2.9)	4.4 (0.9–4.8)	3.5 (0.8–3.8)
**Education level**								
High school	2.5 (0.8–2.9)	1.6 (0.4–1.9)	3.3 (0.8–3.7)	2.8 (0.7–3.1)	3.4 (0.9–3.8)	2.5 (0.8–2.8)	3.7 (0.9–4.1)	2.6 (0.9–2.9)
College	2.8 (0.7–3.1)	1.8 (0.8–2.2)	3.5 (0.9–3.9)	2.2 (0.6–2.5)	4.1 (0.9–4.5)	3.5 (0.9–3.8)	4.1 (0.9–4.5)	3.2 (0.7–3.7)
Graduate	2.9 (0.8–3.2)	1.6 (0.6–2.0)	3.8 (0.9–4.1)	2.9 (0.7–3.2)	4.5* (2.9–4.9)	2.8 (0.7–3.2)	5.5* (3.8–5.9)	4.1* (3.9–4.7)
Post–graduate	3.2 (0.8–3.6)	2.4 (0.9–2.8)	4.2 (0.9–4.5)	2.8 (0.7–3.1)	4.7 (0.9–5.0)	3.1 (0.8–3.5)	5.8* (3.9–6.2)	4.4* (3.5–4.8)
**Occupation**								
Students	2.2 (0.7–2.7)	1.5 (0.6–1.9)	2.5 (0.8–2.9)	1.3 (0.5–1.7)	3.3 (0.9–3.7)	2.6 (0.8–2.9)	4.1 (0.9–4.5)	2.7 (0.7–2.9)
Employed	2.4 (0.8–2.7)	1.9 (0.7–2.2)	2.8 (0.9–3.2)	1.8 (0.7–2.1)	3.5 (0.9–3.9)	2.8 (0.8–3.1)	4.3 (0.9–4.5)	3.5 (0.8–3.9)
Unemployed	2.7 (0.9–3.1)	2.4 (0.6–2.9)	3.2 (0.9–3.7)	2.7 (0.8–3.1)	4.5* (3.8–4.9)	3.4* (2.7–3.8)	4.7* (3.5–5.1)	3.7* (2.9–4.1)
**Family system**								
Joint	2.5 (0.7–2.9)	1.9 (0.5–2.3)	3.5 (0.9–3.8)	2.5 (0.9–2.8)	4.4* (3.2–4.7)	3.4* (2.7–3.7)	5.5* (4.8–5.9)	4.2* (3.74.5)
Nuclear	2.7 (0.9–3.1)	2.5 (0.7–2.8)	3.9 (0.9–4.3)	2.8 (0.9–3.3)	5.2* (4.5–5.7)	4.5* (3.5–4.8)	5.8* (4.5–6.2)	4.5* (3.7–4.9)
**Income**								
Less than 30K	2.6 (0.8–2.9)	2.1 (0.6–2.5)	3.5 (0.9–4.2)	2.3 (0.7–2.7)	4.5* (3.5–4.8)	3.7* (2.9–4.1)	5.5* (4.5–5.8)	4.2* (3.5–4.7)
30–60K	2.5 (0.9–2.8)	1.7 (0.8–2.1)	2.5 (0.7–2.9)	1.8 (0.7–2.2)	3.5 (0.9–3.8)	2.5 (0.8–2.9)	4.5* (3.7–4.8)	3.5* (2.9–3.9)
61–90K	2.4 (0.8–2.8)	1.5 (0.5–1.9)	2.1 (0.7–2.5)	1.7 (0.6–2.1)	3.1 (0.9–3.5)	2.3 (0.8–2.7)	3.5 (0.8–3.8)	2.8 (0.9–3.1)
Above 90K	2.2 (0.8–2.6)	1.2 (0.5–1.8)	1.8 (0.5–2.1)	1.5 (0.7–1.8)	2.8 (0.9–3.2)	2.1 (0.8–2.5)	3.2 (0.9–3.7)	2.4 (0.7–2.7)
**Marital status**								
Married	1.8 (0.7–2.2)	1.7 (0.6–1.9)	3.1 (0.8–3.5)	2.5 (0.7–2.8)	4.5 (0.9–4.8)	3.3 (0.9–3.7)	5.4 (0.9–5.9)	3.8 (0.9–4.2)
Single	2.1 (0.6–2.5)	2.5 (0.7–2.9)	3.5 (0.9–3.9)	2.7 (0.7–3.1)	4.8 (0.9–5.2)	3.7 (0.8–4.1)	5.6 (0.9–5.9)	4.5 (0.8–4.9)

Reference group: Not feeling this way at all; ^*^indicates significant at 0.01 level.

## Discussion

This research has addressed an identified gap in the literature whereby little has been written about shame, guilt, and their relationship to inpatient polysubstance abusers. This study aimed to determine the correlation between shame and guilt-activation among polysubstance abusers hospitalized in a rehabilitation center. The study also explored whether the people with SUD reported higher shame and guilt scores than the control group. We were also interested in how sociodemographic characteristics might be associated with shame and guilt scores among people with SUD and control groups.

The study was restricted to male participants, given the fact that SUD is significantly more prevalent in men where the study was conducted, and whether the findings of this study are generalizable to female populations needs further exploration (i.e., 78% of those with a SUD in Pakistan were male in 2013.

The findings from this study have revealed a strong connection between shame and guilt scores among polysubstance abusers. The findings supported the first hypothesis and suggested that polysubstance abusers who experience a higher level of shame also experience a higher level of guilt. This relationship is possible because people with SUD take drugs to cope with negative self-conscious emotions, including shame and guilt. The more they take drugs to relieve their shame and guilt-based feelings, the more they experience shame and guilt. Using drugs is the only quick way to get short-term relief from these painful emotions (20). Such acting out then causes negative consequences and creates even uncomfortable feelings of shame and guilt.

The findings are consistent with other studies which show shame-activation to be positively associated with guilt-activation on alcohol dependence among university individuals (7, 9, 10, 21, 22). According to cognitive theories of addiction, relying on drugs as a mood adjuster can lead to the development of substance use disorder (23). Shame and guilt activations may be responsible for inner judging voices that may need to be silenced with maladaptive behaviors.

Additionally, as we expected, healthy individuals will likely have lower shame and guilt scores compared to people with SUD. We further compared the shame and guilt scale between healthy individuals and people with SUD. We found that people with SUD reported a higher tendency to experience shame and guilt-activation than healthy individuals. So, the findings also confirmed the second hypothesis. It is thought that the reason for this is the efforts of people with SUD to get rid of the situation are in vain, and the negative feelings caused them to finally surrender to addiction. This study corroborated the previous studies (22, 24, 25), which showed that guilt and shame might contribute to alcohol and drug addiction. The authors proved that the people with SUD felt more guilt and shame than the individuals from the control group. The results of another study in which 60 alcoholics and 40 healthy people were compared showed that the alcoholics had significantly higher levels of shame and guilt (26).

The studies conducted on the Turkish sample examined the feelings of shame and guilt in individuals who use and do not use alcohol, and they proved that the guilt and shame levels of people with alcohol problems are higher than those who do not have alcohol problems (27, 28). Similarly, comparing the degrees of guilt and shame between the control group and alcoholics in remission, it was found that the intensity of these feelings was higher in alcoholics (29).

Regarding sociodemographic factors, we observed significant mean differences in shame and guilt across age groups between people with SUD and healthy individuals. The findings presented here provide two clues, participants from the group with SUD reported higher shame and guilt scores in all age groups, and the control group reported a lower level of shame and guilt. Results suggest participants in high-age groups experience a higher level of shame and guilt. Consistent with the previous study (11) which proved that in late adulthood, individuals experience more emotions of shame and guilt. Furthermore, the literature supports that in late adulthood person's feelings of shame and guilt are followed by emotions such as regrets in life (30). In later life, it is difficult for an individual to fix these strong emotions like guilt (31). Therefore, it can be stated that guilt greatly affects the life satisfaction of older individuals as compared to younger ones.

Results further revealed significant mean differences in education level within groups. Findings demonstrated that educated people feel more shame and guilt while suffering from addiction. Higher shame and guilt might occur at a high level of education. Socio-emotional Selectivity Theory (32) Suggested that when an individual achieves maturity level along with greater knowledge as a result of educational level, they start perceiving that time is running out and become more present-focused as compared to future and more goal-oriented. It also changes the person's emotional processing of information, ultimately reducing negative emotional experiences, and they can interpret their emotions more appropriately and positively. It is also assumed based on the above theory that while attainting treatment and awareness sessions regarding drug addiction side effects, how it affects the person's life, and how they better cope with life stressors, it may change their emotional processing and further they can be able to interpret their life experiences more positively and cope more effectively.

Further, results suggested unemployed participants experience greater shame and guilt than other occupation ranks. The recent research on a large clinical population revealed that unemployment status, older age, and lack of social support are related to suicidal ideation in gambling disorder, shopping disorder, gaming disorder, and sex addiction, and these disorders are characterized by higher levels of shame and guilt feelings (33–35). In addition, polysubstance abusers may have less family support, loss in their business, or be kicked off from jobs, and after all, they spend all their savings in buying drugs. Maybe that's why people with SUD having unemployment status experience higher feelings of shame and guilt. Financial problems, social isolation, and feelings of failure that frequently come with unemployment may contribute to the increase of shame and guilt feelings.

### Limitations and future directions

Our study has several limitations. Firstly, due to the cross-sectional research design, it would be difficult to comment on the direction of relationships among these variables in the current study. However, future research must be based on longitudinal design to investigate whether the degree of shame and guilt change over time. Secondly, we used one scale to measure all types of guilt; future studies might use different scales for each type of guilt e.g., situational, standards, and chronic guilt. Moreover, in the present study, other psychiatric disorders remain as an exclusion criterion; future studies should investigate the joint influence of polysubstance dependence and comorbidities (e.g., post-traumatic stress disorder (PTSD), anxiety, and depression) on shame and guilt-activation. Finally, our whole sample consisted primarily of men. Future research should examine whether shame and guilt affect men and women differently.

### Implications

From the study results, one possible implication for clinicians is the importance of evaluating people with SUD for their shame and guilt levels. Clinicians may help patients differentiate between shame and guilt, particularly by noting the motivating potential of guilt and the more extreme negative significance of shame. The present study's findings provide more credence to Dearing et al. (24) contention that it is critical and imperative to distinguish between shame and guilt-activation when the constructs are considered in contexts related to drug use research and treatment. According to the results of the current study, shame-prone people may benefit from learning how to deal with negative emotions in clinical settings without resorting to drugs, which could lead to drug dependence. Further, current research added to the body of knowledge on sociodemographic characteristics by providing evidence that polysubstance users who are older, more educated, unemployed, from nuclear families, and have lower incomes feel more shame and guilt.

## Conclusion

We found a strong relationship exists between shame and guilt activations among inpatient polysubstance abusers. The findings of this study confirm that polysubstance abusers experience a higher degree of state shame and guilt than healthy individuals. Further, polysubstance abusers with older age, higher education, unemployment status, nuclear family system, and low income, experience higher levels of shame and guilt.

## Data availability statement

The raw data supporting the conclusions of this article will be made available by the authors, without undue reservation.

## Ethics statement

The studies involving human participants were reviewed and approved by the Ethics Board Committee of the Department of Psychology, Wuhan University, China. The patients/participants provided their written informed consent to participate in this study.

## Author contributions

NA, MI, and SM: conceptualization and methodology. NA, MI, YY, SM, MT, and RY: validation and visualization. NA, MI, MN, and RY: software. NA, MI, SM, and MT: writing—review and editing. MI, SM, and MN: formal analysis. YY, SM, MN, and RY: investigation. NA: funding acquisition. MI: roles/writing—original draft. YY: project administration, resources, and supervision. MN: data curation. All authors contributed to the article and approved the submitted version.

## Funding

This research was conducted under the Mianyang Normal University's scientific research promotion project The effect of Praise and Academic Motivation on Social Identity on college adaptation among College Students: A Cross Cultural Comparison between China and Pakistan (QD2020B04).

## Conflict of interest

The authors declare that the research was conducted in the absence of any commercial or financial relationships that could be construed as a potential conflict of interest.

## Publisher's note

All claims expressed in this article are solely those of the authors and do not necessarily represent those of their affiliated organizations, or those of the publisher, the editors and the reviewers. Any product that may be evaluated in this article, or claim that may be made by its manufacturer, is not guaranteed or endorsed by the publisher.
